# A Qualitative Exploration of the Impact of Increasing Criminalization on Domestic Violence Support Workers: Nonfatal Strangulation as a Case Study

**DOI:** 10.1177/10778012241289422

**Published:** 2024-10-24

**Authors:** Leah S. Sharman, Heather Douglas, Robin Fitzgerald

**Affiliations:** 1 Faculty of Law, 2281 University of Melbourne, Melbourne, Victoria, Australia; 2 Faculty of Humanities and Social Sciences, 1974 University of Queensland, Brisbane, Queensland, Australia

**Keywords:** strangulation, choking, domestic violence, criminalization, support workers

## Abstract

Specialized nongovernmental domestic violence (DV) services provide critical support to victim/survivors. This research draws on 14 semistructured focus groups with 27 DV support workers to examine how expanded criminalization impacts support workers’ roles using a case study of a 2016 nonfatal strangulation offense legislated in Queensland, Australia. Our results describe a lack of governmental support intersecting with increased complexity and higher workload burden resulting from expanded criminalization. Expansions to criminal law need to account for and critically assess the full system impact of new DV legislation and the added burdens placed on the pivotal third sector of DV services.

Recent decades have seen significant law reform and increasing criminalization of domestic violence (DV) in Australia (Braaf, 2008; Woodlock et al., 2023) and globally (Danis, 2003; Fentiman, 2022; Goodmark, 2021). These efforts have sought to address acts of DV through the introduction and implementation of criminal laws, which center police and legal systems as primary intervention systems to support and protect victim/survivors of violence (Danis, 2003; Fentiman, 2022; Queensland Government DJAG, 2021). Criminal laws focused on DV have included more punitive criminal responses to breaches of civil protection order conditions, assaults (including sexual assault), damage to property, threats, intimidation, coercive control, stalking, exposure of children to DV, economic abuse, emotional abuse, and pet abuse carried out in the context of domestic and family violence (Australian Government, 2009; Queensland Government, 2021, 2022). In the context of this expanding criminalization, we often consider the impact on the operations of police and courts, but rarely, the pivotal third sectors (Lyons, 2001). In the case of DV, that includes support workers within the nongovernment DV support sector. In this article, we focus on these service workers in Queensland, Australia to understand what consequences new DV criminal laws have on their work to support victim/survivors, using the introduction of the nonfatal strangulation offense as a case study.

## The Third Sector

The third sector (also termed “community sector”) describes the many organizations that exist outside of the duality of government and for-profit business (Lyons, 2001). They operate primarily within the not-for-profit space, largely independent of government, and often with a strong volunteer and collective action basis. These enterprises encompass an extremely diverse array of activities and include trade associations, child-care, social and mental health support services, disability foundations, aged-care services, and churches, to name just a few.

The third sector provides a substantial contribution to communities globally. However, they are constantly undergoing major and often highly disruptive changes due to shifts in policy and government support across sectors (Lyons, 2001). In many cases, these changes result in increasing demands on services, with the same or fewer resources available to them, impacting their equity, productivity, and effectiveness (Smith, 2002). For DV services, these changes are often related to modifications of existing legislation and the introduction of new legislation regarding responses to DV.

### DV Services: A Crucial Third Sector

DV services aim to work with victim/survivors to increase their safety, providing referrals and assistance as part of “safety work” (Woodlock et al., 2023). This can involve safety planning, referrals to shelter services, legal information (Afifi et al., 2008; Coker et al., 2005; Macy et al., 2009), and assistance with criminal legal processes and interactions (Department of Child Safety, Youth and Women, 2019; Douglas, 2021; Mundy & Seuffert, 2021). They also provide a safe space for victim/survivors to regain control over their decision-making and actions to increase their equal participation in society, empowerment, and sense of well-being as part of “freedom work,” which can include counseling and connection to the community as part of this process (Sullivan, 2018; Woodlock et al., 2023). However, services’ capacity to undertake freedom work has slowly been decentered over the decades in favor of safety work, which has grown in part to support state criminalization of DV (Meyer, 2011; Woodlock et al., 2023).

Among many Western countries, DV services are now built into social policy and form a vital part of the response to DV (Ekström, 2018; Sullivan, 2018). They are typically nongovernment organizations, located in the community, and in the Australian context, they utilize a mixture of volunteer and paid staff providing substantial wrap-around support to victim/survivors. Their roles encompass a wide range of services including telephone helplines, advocacy (e.g., for child safety, housing, police, and courts), counseling, support groups, temporary housing, and protection, and can include behavior change programs for men and perpetrators. They deliver diverse support and assistance centered on feminist, survivor-led practices (Woodlock et al., 2023), with each interaction requiring substantial emotional labor. That is, not simply the management of their own emotions, but the adoption of approaches to manage the emotions between themselves and victim/survivors (Hochschild, 2012). We refer to the combination of diverse roles supporting victim/survivors as “support workers.”

Disclosure and reporting of DV is challenging for many victim/survivors of abuse. Barriers often include fear of consequences, such as fear of children being removed; negative judgments from family, friends, and the person they disclose to; fearing they will not be believed; and fearing intervention by authorities will increase the risk of abuse (Heron & Eisma, 2021; Meyer, 2011). Although police and health workers are often on the front line responding to domestic violence, specialized DV support services are often sought after serious episodes of violence or near-death experiences, such as strangulation (Meyer, 2010, 2011; Petersen et al., 2005).

In Australia, 64% of people who experienced DV reported seeking advice or support after their most recent experience of violence, with victim/survivors reporting that DV services, including counselors, support workers, and helplines, were the second most common source for support and advice after informal supports (i.e., friends or family; AIHW, 2022). Importantly, they were also the most common formal help-seeking resource, over other health practitioners or legal avenues, including police. While these avenues for help seeking reflect the Australian context, a similar hierarchy for help seeking is maintained in other countries with developed DV sectors (Evans & Feder, 2016; Robinson et al., 2021). Thus, in Australia and elsewhere, as one of the key frontline responders, and often the only formal services adequately prepared to address DV, DV services confront the full spectrum of abusive behaviors, including physical abuse, sexual violence, child safety concerns, and controlling and coercive behaviors.

## DV Support Work in the Context of Rising Criminalization

Globally, states have gradually widened the range of DV harms for which a criminal sentence can be imposed (Blagg et al., 2018; Fitz-Gibbon & Walklate, 2021; Goodmark, 2018, 2021). Scholars have laid out the negative consequences of the rise in the rate of arrests, convictions, and imprisonment for DV perpetrators in Australian states and territories, and particularly how this rise has affected First Nations communities (Douglas & Fitzgerald, 2018; Hobson et al., 2023; Macoun et al., 2021). Consequences have also been acutely felt among victim/survivors, with this rise in criminalization not equally matched with provisions for support through the criminal legal process. Australian victim/survivors of sexual violence have reported feeling uninformed about the process and trial decisions, exposed to stigmatization and retraumatization, and feeling disillusioned with justice responses (NSW BOCSAR, 2023; Women's Safety and Justice Taskforce, 2022). While research has questioned the relationship between criminalization and the consequences for victim/survivors, the question of whether, or how, increased criminalization affects support workers is yet to be examined.

DV services now play a key role in government responses to criminalization (Ekström, 2018; Mundy & Seuffert, 2021; Sullivan, 2018). In an every-day context support workers’ interactions with the criminal legal system involve assisting victim/survivors with initial reporting to police; crisis intervention, support, and safety-panning for people referred by police after their attendance at DV incidents; and support for victim/survivors by specialized court support workers to assist with the complexities of court proceedings that can include assistance with applying for private protection orders or varying existing protection orders that may or may not have been a police application (Department of Child Safety, Youth and Women, 2019; Douglas, 2021; Mundy & Seuffert, 2021). In trends reflected globally, the services of this third sector have been increasingly in demand with rising incidents of DV being reported and a greater need for support services following the COVID-19 pandemic and rising economic hardship (Campbell, 2021; Pollard, 2021; Uzobo & Ayinmoro, 2023). Along with this greater need from victim/survivors reporting and demand for support confronting DV, there is an expectation that support workers are able to add to their ever-increasing workloads by adapting and incorporating legislative changes into their practice (Lyons, 2001).

## The Nonfatal Strangulation Offense

Strangulation is a common and dangerous form of gendered violence with women up to 13 times more likely to experience this type of violence than men (Patch et al., 2018; Thomas et al., 2014). When the act is nonfatal and occurs within the context of DV, women are at an seven-fold greater risk of becoming a victim of homicide or very serious harm in the near future (Glass et al., 2008). Furthermore, this type of violence can easily be fatal, or cause a range of injuries, although they are often not visible (Sharman et al., 2023). In recent recognition of these risks and the dearth of visible injuries, specific offenses of strangulation in DV have been introduced in a number of jurisdictions globally (Edwards & Douglas, 2021). However, this growing recognition has been slow to translate to knowledge in the wider community (Sharman et al., 2024) and the health sector (Pritchard et al., 2017; Queensland Health, 2017).

In Queensland, acknowledgment of the injuries associated with strangulation and increased risk of death led to the introduction of the non-fatal strangulation offense (Queensland Government, 2021), s315A, into the Queensland Criminal Code—“Choking, Suffocation or Strangulation in a Domestic Setting” with a maximum penalty of 7 years imprisonment. The introduction of this offense meant strangulation incidents as part of DV did not rely on a visible injury to be charged (e.g., as required by the offense of Assault Occasioning Bodily Harm (*Criminal Code Act 1899* s339)) and elevated it above comparatively minor charges such as Common Assault (*Criminal Code Act 1899* s335).

Following the introduction of the offense, 97% (929) of matters that proceeded to court were sentenced for offenses of nonfatal strangulation in the first 4 years (Sharman et al., 2022). Of those sentenced, where strangulation was the most serious offense, 99% of offenders pleaded guilty. Sentences were an average of ∼2 years imprisonment and 54% of offenders were remanded in custody without bail over an average time period (date of offense to sentencing) of 1 to 1.5 years. However, around half of people charged actually proceeded to adjudication and many strangulation charges were downgraded (Sharman et al., 2022). Furthermore, like other offenses such as rape and sexual assault, strangulation within DV is likely to be widely underreported to police by victim/survivors (i.e., part of the “iceberg” of DV), resulting in no charges at all (Douglas & Fitzgerald, 2022; Gracia, 2004).

The introduction of the nonfatal strangulation offense in Queensland provides a recent case study for examining what impact a new DV-related offense has on one of the frontlines for responses to violence, DV support workers. This research used qualitative focus groups with support workers and assessed (1) if the introduction of the NFS offense has impacted how they engage with victim/survivors disclosures of strangulation, (2) the challenges posed to support victim/survivors with the new offense, and (3) support workers’ experiences of engaging with criminal law for NFS.

## Method

### Participants

Participants all worked with or managed components of services for victim/survivors within community-based, nongovernmental services. Those working primarily with perpetrators were excluded for analysis as perpetrator programs are often utilized at the end of the criminal legal process. Focus groups included 27 DV support workers overall, comprising 16 victim/survivor specialists who described themselves as counselors, caseworkers, intake specialists, court support workers or advocates, and social workers. Participants also included three service managers; six child and family counselors or child safety workers who provide some overlapping support alongside victim/survivor specialists, as well as clinical assessment, psychosocial therapeutic and educational support to children and families; and two men's behavior change program facilitators who were also victim/survivor counselors. Victim/survivor specialist roles included some or all aspects of: case management for victim/survivors, conducting risk assessments, crisis support, and safety planning, referral to and coordination of different services, monitoring and regular review of clients’ progress, and supporting victim/survivors and families into longer term housing and supports. Court support workers separately assisted with protection order applications, provided support for victim/survivors to engage with police or other legal services, and supported victim/survivors through court proceedings.

All participants delivered similarly funded and structured support services (Queensland Government, 2018) through eight providers to victim/survivors in Queensland across nine urban, regional, and rural areas. On average, participants had worked in their roles for 2.5 years (*M *= 30.70 months, *SD *= 25.73), ranging from 3 months to 9.5 years, for the 23 participants who provided this data.

### Focus Groups and Analysis

Fourteen semistructured focus groups took place online via Zoom from June to October 2020, exploring support workers’ knowledge and responses to strangulation, perceptions of how strangulation and the legislation affects victim/survivors and perpetrators, depending on their role, and their experience of the criminal legal system in response to charges of nonfatal strangulation. Focus groups typically included two to four participants and were led by two authors. One participant was interviewed one-on-one due to the absence of other attendees. Focus groups were an average of 67 min, ranging from 41 to 90 min in length.

Thematic analysis using an iterative process was employed to examine the data using a combination of essentialist and social constructionist approaches (Terry et al., 2017). Analysis followed a well-established iterative six-phase procedure described by Braun et al. (2019). Analysis of the data was led by the first author with other authors contributing to discussions of theme meanings and label refinement. The authors all identify as women who have had varying personal and professional interactions with the domestic violence sector and have backgrounds in social and health psychology (LS), law and criminology (HD, RF), and sociology (RF).

### Ethics

Ethics approval for this research was granted by the University of Queensland Human Research Ethics Committee (2020000558). All participants provided written and/or verbal consent before the interviews.

## Results

**Figure 1.**
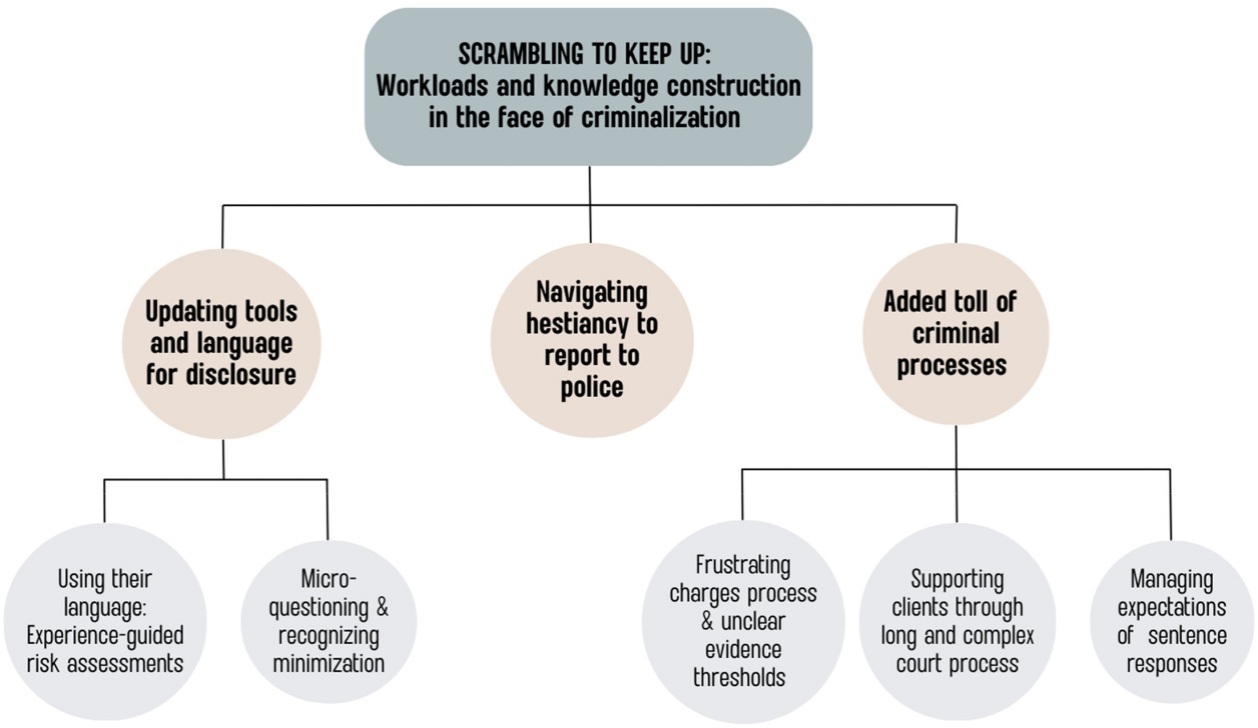
Thematic map of domestic violence support workers’ perceptions of strangulation and the offense.

Thematic analysis generated one overarching theme from the data—Scrambling to keep up: Workload complexity and knowledge construction in the face of criminalization. This overarching theme informed three subthemes on the skills support workers employ to understand experiences of strangulation, how they navigate reporting to police, and the added labor needed to navigate the criminal legal system when dealing with strangulation. Each of these themes contains a range of intricacies regarding the specifics of navigating new operational conditions around the offense. These adjustments affected how DV workers interacted with many different services within and outside of the DV sector, and between DV support workers and victim/survivors. Our results provide a broad overarching view on the dimensions of difficulty reported by DV workers responding to a new criminal offense. Figure 1 provides an overview of the constructed themes.

### Scrambling to Keep Up: Workloads and Training in the Face of Criminalization

Across respondents, it was clear that the introduction of the offense had increased the complexity of support workers’ workloads without any extra on-going government support for training or understanding the offense. Support workers were left to create their own process of knowledge construction and respond to a lack of knowledge across victim/survivors and external services. Although workshops and training sessions for understanding strangulation were available through another nonprofit charity (https://strangulationprevention.com.au), the ability for support workers to attend was inconsistent due to high workloads, cost of attendance, and the minimum full day time commitment usually required. Because of this, training was reported inconsistently across organizations, with some services having had all support workers trained and others only having one or two. Thus, internal peer-to-peer education of colleagues was very common, with the onus on people who had been able to attend external training and education days to share the information informally among those unable to attend:

In the five years, I’ve never had it. I’ve just had some resources shared by colleagues that have been lucky enough to go. So, I would love to go, but it hasn’t… The stars haven’t aligned for me to go. (Counselor 5)

The importance of this internal peer-to-peer information flow from colleagues who had received training was reiterated as integral to the confidence for many to work with victim/survivors who disclosed strangulation experiences. For those who had not attended training, this support helped them to understand risks, consequences, how to ask questions, and know what steps to take with victim/survivors after disclosure:

So, [from my colleague] I have picked up an incredible amount of knowledge and skills about how to ask questions, what's important, what do I need to ask. But if I had been an advocate who was in the counselling team doing things alone… I would have absolutely no idea. (Child safety 2)

Regardless of training, support workers identified a long list of service-generated strategies they needed to employ. Firstly, to identify whether victim/survivors had experienced strangulation, and subsequently how they acted on those disclosures. Across services and roles, support and advocacy included: creating safety plans; education for victim/survivors about the health risks and legislation; referral to health services for health checks but also to facilitate investigation and recording of injuries for evidence; taking photos of injuries for future evidence if needed; making referrals to other relevant services (e.g., child safety and housing); assistance with police reports, court preparation, tempering of victim/survivors’ expectations regarding consequences for perpetrators; and liaising with Queensland Police and courts to assist with communication to victim/survivors around charges and bail:

…we will do at least these things. So, we’ll at least talk about the risk, offer medical assistance, let them know what services can assist, that it's a criminal offence, those sort of things. (Family counselor 1)

Advocacy and education about strangulation was not just internal with colleagues and victim/survivors, but information flowed outward to external services. Many support workers said they provided education about strangulation risk and injuries to police, ambulance services, and general practitioners (GPs). While it was not always clear how education was provided to each of these services, there seemed to be a consistent method for approaching GPs. Because referral to health services after a report of strangulation had become commonplace, many services reported developing a template “Dear Doctor” letter provided to victim/survivors to take with them to appointments. This letter identified that the person had been strangled, the types of injuries they may have, and best practice regarding scans to identify internal injuries:

A little print out of a little A4 sheet that women can take to a GP that says ‘I have experienced strangulation, evidence base says a CT scan is best practice, here are some locations in our local area, can you please make a referral to me’. And then it means that the evidence is there for the GP and they can make that referral easily and it's not confronting for the GP either. (Child safety 2)

This quote and the one below suggest that health professionals sometimes feel confronted by, discount, or ignore information presented by victim/survivors, to the detriment of these victim/survivors. Thus, more work has been invested in ensuring health professionals are aware of the risks of strangulation so they take victim/survivor accounts seriously and make the requisite referrals:

I had feedback from one of their advocates that they took that information to the GP, but the GP was still like, ‘I’ve never heard of these people, what is this, we don’t do that here’. And so then she had to go to a different GP and then get a different response. (Court support worker 2)

Overall, the number of steps and increased complexity of the workload needed to respond to strangulation disclosures highlighted how an ad hoc process of knowledge construction and dissemination was required to keep up to date with legislation change. Further adding to this burden was the high frequency of strangulation disclosures heard by support workers, highlighting how pervasive this workload has become since the introduction of the offense:

I think in the risk assessments I’ve done, this week… I think every single one of them had identified it. So, 18-year old's, 40-year old's, 60-year old's. I think it's an experience that's across the board and across demographics, for women that we’re doing risk assessments for. With many of the women, the strangulation has occurred recently, in the last couple of days, or the last couple of weeks or months. (Intake specialist 1)

#### Updating Tools and Language for Disclosure

##### Using Their Language: Experience-Guided Risk Assessments

Since the introduction of the offense and the availability of training, several services detailed that the way they asked questions about strangulation had gradually changed, going beyond typical items in validated risk assessment tools. Participants identified that questions on risk assessments often simply had a check box next to whether “strangulation” had been identified. Yet, rarely did victim/survivors identify their experiences using the words “strangulation,” and even when given an explanation, rarely felt that it applied to them. This resulted in some support workers adjusting their risk assessments to reflect knowledge learned from a combination of training and experience with victim/survivor disclosures:

[Our risk] assessment was specifically changed and developed so that it would be easier for workers to ask that without perhaps just saying ‘have you been strangled’… the staff felt that there was the gentler way, more narrative way of doing the risk assessment. And so, they used their skills and experience to tweak the question so that they were getting the better answers that they thought were really reflecting the experiences of the clients a bit better. (Manager 2)

##### Microquestioning and Recognizing Minimization

Having victim/survivors disclose their experiences of strangulation was not a simple linear process. Questions about strangulation were described as requiring appropriate timing—being raised after a strong rapport was built, sometimes taking multiple sessions, and tailoring the language to what each victim/survivor needed to be able to explore the violence:

Unless I'm asking it in different language and providing different opportunities to explore the violence, I don't think many of the women would actually go, yes, I have been strangled. It's how you ask the question and how you present the question… (Counselor 6)

How questions about strangulation were asked was commonly described as an indirect and non-technical process, coined “microquestioning” by one support worker. This involved practiced and skillful use of small and incremental questions to give victim/survivors the space to slowly reveal the complete picture of strangulation experiences and their consequences:

But then when you drill into finding out how serious it was, you find out, yes, that they did lose consciousness, that they did see stars and couldn’t breathe, and you get a whole lot more rich information. (Social worker 2)

Victim/survivors were universally perceived by support workers to minimize their experience of strangulation with few having knowledge about the existence of the offense. There was a consensus among these descriptions that strangulation was not considered by victim/survivors as having consequences as serious as other types of physical violence:

[a woman] who was being strangled, two to three times per week, to the point where she was passing out, for 12 months. And she was like, I just felt lucky that he wasn’t hitting me. She did not consider it as physical violence. It just didn’t fall in that same category. (Case worker 1)

Victim/survivors’ perceptions and limited understanding of the severity of strangulation impacted how support workers discussed it, as asking about broader experiences of physical violence may not lead to disclosures of strangulation. Thus, microquestioning and asking about “holding,” “pushing,” “grabbing,” and placement of hands around the neck and shoulders were described by all support workers as necessary to identify strangulation in a language victim/survivors could recognize. These also included using the term “choking” rather than strangulation, and asking whether there was a time they struggled to breathe because of these actions:

This is a massive thing to have in the back of your mind… you may ask, has he strangled you, and he may well have done one of those acts, but she doesn’t think that it's strangulation. So, it's really about trying to unpack or being more specific when you are asking them. So, ‘has he ever put his hands around your throat?’ (Counselor 2)

Memory loss was identified as a particularly challenging barrier to asking about and understanding strangulation experiences. Support workers described victim/survivors’ difficulty in relaying the strangulation event and forming a cohesive narrative about the violence:

…there's elements of memory loss associated with it, which we know is a really common thing. They’re like, oh, yes, it did happen. But they can’t remember the specifics of it. They may not remember what happened directly before it. And so, it feels like this thing, that because it's so hard to describe and so hard to kind of detail what's happened, they’re like, oh, well, that's not something that's as important. (Case worker 1)

#### Navigating Hesitancy to Report to Police

Support workers acknowledged general reluctance for victim/survivors to report to police due to shame and embarrassment (particularly where strangulation occurred during sex), wanting to continue the relationship, or wanting to “move on” and avoid added stress. However, they also identified the difficulty of navigating victim/survivors concerns that their experience would be taken seriously and believed in the absence of any marks, bruising, or other evidence:

If there are no marks, it's like…‘What evidence do I have? I don’t have any evidence.’ So even if I want to report this, what am I going to say? And to explain how it happened, especially when it happens during sex, can be incredibly hard for a woman. (Counselor 4)

Furthermore, there were multiple accounts of victim/survivors resisting or fighting back. These actions of survival meant injuries were sustained by perpetrators and, due to the nature of strangulation injuries, few to no injuries on themselves. For support workers this came with the added complexity of navigating how they could assist victim/survivors to report without the risk that they might be charged with an offense instead of the primary perpetrator of violence:

One of the biggest issues that we’ve faced is that defensive wounds of women reaching out to scratch or to pull an offender's hands off them or biting their hands or anything they can to survive the strangulation. (Counselor 6)

Support workers reported that some victim/survivors, such as First Nations persons and people in rural areas, felt underserved or unprotected during current or previous DV incidents by police and other services set up to protect them. Thus, support workers discussed that they had to contend with a hesitancy to disclose strangulation experiences, and a loss of trust in the system or sense of defeat in reporting these experiences:

Those responses that we see so often from the police or the doctor, women just stop even mentioning it sometimes when it comes to strangulation a little bit as well because it's been dismissed so many times before. There's nothing in their world that's shifted that would make them believe that anything would be different. (Counselor 5)

Support workers also commented that many victim/survivors felt that reporting to police would not lead to sufficient protection and safety from harm from the perpetrator:

I guess that feeds back into why some women are so fearful of going through with reporting and that because if there's no guarantee that he's going to be either remanded in custody or there's any other kind of repercussions and that even while they were awaiting that court date, and that's… That, I guess, increases their safety [risk] in that time. (Counselor 2)

This system-based hesitancy and mistrust could at times lead to barriers in victim-survivors confiding in support workers. However, support workers contended with this by centering victims-survivors and maintaining their safety and preferences. In this way, providing all options and information about reporting processes was part of trust building to ensure victim/survivors felt heard, protected, and a confidence in reporting strangulation incidents to support workers in future where police involvement was a choice:

I think they’d be open to discussing [strangulation incidents] with our service, because we’ve made it clear that we don’t take anything further unless they wanted to, we don’t disclose that information to police without their consent… Yes, so, they would probably tell us if something happened in the future, but yes, I think definitely it [concerns about child protection investigations] could influence their decision to report to police. (Child safety 2)

#### Added Toll of Criminal Processes

Support workers expressed a variety of ways that supporting victim/survivors through criminal legal interactions and processes increased demands on their roles, often involving extra emotional burden. This included both increased personal frustration and added emotional labor required to help manage victim/survivor's experiences and expectations.

##### Frustrating Charging Process and Unclear Evidence Thresholds

Support workers perceived that there was a lack of clarity around knowing what threshold of evidence was required for police to proceed with charges. At times, they described victim/survivors as having one or more significant and visible injuries, documentation by medical professionals, witnesses, and even video evidence of the strangulation. Despite this, support workers described being told frequently that this evidence would not be enough or meet the threshold to proceed with charges:

I [asked] are you doing anything about this strangulation that you’ve put in your referral to us? And they came back and just were like, no, it doesn’t meet our threshold, that wouldn’t be what we consider to be a strangulation. And when I finally got the full copy of the cross order, this was the most articulate description of a strangulation I’ve ever seen. (Case worker 1)

Feelings of frustration from police responses were compounded when support workers felt their workloads were increased because the responsibility for pursuing evidence collection and client safety appeared to be on victim/survivors and themselves, rather than police, to progress strangulation charges:

Yes, and we encourage it and help them to find ways to gather things because we know how handy it is. If you have evidence we have more chance of getting [*protection orders*] and enforcing them on breaching perpetrators. And yes, because a lot of the times it is up to the women to gather their own evidence to help protect themselves, sadly. (Family counselor 3)

Support workers recounted numerous instances where they perceived police had not appropriately collected or assessed evidence or statements, potentially due to some police not understanding the types of injuries typically sustained or how to respond to allegations of strangulation:

And often … they will mention that they have been strangled and the police are looking for any marks on their neck and if they can’t see anything then it's often dismissed because they can’t visually see anything. So, they don’t have that understanding. It's not very often that we hear that the police have actually asked any additional questions… (Manager 3)

Police reports downplaying the event were also described as occurring often. This minimization from police was seen as leading to a lack of evidence collection or follow-up, and therefore failure to charge perpetrators for strangulation:

And even in one of our police-assisted referrals today, and I see this a lot, is that they’ll kind of mention strangulation but the police will then *downplay it*… Both hands around the neck, oh but he didn’t squeeze, so it's okay. (Court support worker 1)

Support workers who had advocated for and assisted victim/survivors to report to police or seek medical care, felt frustrated and dismissed when they were met by what they perceived to be unsatisfactory or indifferent responses regarding victim/survivors’ experiences of serious violence. This included where police failed to apply for protection orders on behalf of victim/survivors, which in Australia is the more common and most successful pathway to having a protection order granted (Fitzgerald & Douglas, 2020). The failure to protect victim/survivors meant that, where possible, support workers had to fill in the gaps left by police and were even expected in some circumstances to take over this role:

I had a [client] who…took [a video of her strangulation to] a rural police station and they were like’ sorry, we’ve got to have this conversation out on the footpath because we’re closed on Sundays.’ And then she was like ‘what do I do? Here's this video of me being strangled, I’m really scared for my safety.’ The police officer said ‘well, do you have a DV worker? Go and tell them and get a private application because we’re closed and this was two days ago and you didn’t come here straight away.’ I ran screaming to our court advocate and got a [protection order] with full conditions on it. (Family counselor 3)

For many support workers, these experiences shaped their future expectations with reporting and encouraging victim/survivors to report. Some communicated that they felt the emotional and physical energy required for victim/survivors to report was not worth it.

But for the ones that do want to [report to police], it feels like you can’t even encourage that, because you know that there's no point. Eventually, it becomes not an exercise that's a useful use of her time or useful use of her energy. (Intake worker 1)

##### Supporting Clients Through Long and Complex Court Process

In contrast to lower court sentencing processes, such as for common assault, criminal court processes for the more serious charge of non-fatal strangulation were described as marred with fractured communication and information. At times, support workers described a protracted process and burden of information finding where each avenue resulted in no outcome, resulting in victim/survivors feeling isolated from the court process:

There's been times that I’ve tried to get extra information for [victim/survivors] about where the proceedings are up to, and I can’t get that information at times to give back to [them]. It can be disjointed when you talk to the police and they don’t know and they say, ‘go to the court’, and you talk to the court and they send you to go to [the Office of the Director of Public Prosecutions], and you go to [the Office of the Director of Public Prosecutions] and then they don’t really respond. So, it can be really, really tricky for victim/survivors to know what's happening when they’re there for criminal charges. (Social worker 1)

When strangulation charges were dropped or downgraded to lower charges, victim/survivors were not privy to these conversations or provided updates. Support workers discussed that the lack of communication around this process caused significant anxiety and stress for victim/survivors as these changes were perceived to impact both victim/survivors and support workers’ ability to sufficiently assess and plan for their safety.

So, I think not having a lot of support through the police and through the courts makes it difficult sometimes for [victim/survivors] to know what's happening. And that information is really crucial to their safety and their own assessments of safety at that time. Because what happens, what the result is of those charges can really impact their safety. (Social worker 1)

For charges that continued, the time taken for charges to be laid through to finalization of sentencing was seen as a barrier for victim/survivors to continue as witnesses. Support workers felt that many victim/survivors wanted to move on with their lives and this frustration and emotional toll was challenging for support workers to navigate. This was described by one participant as leading to hostile witnesses:

Again, for many women, it's just like ‘it's too long. It takes too long, I’m over it, I just want to get on with my life’. And I guess some of the conversations I’ve had, particularly with the [police] inspector here will often talk about women presenting as hostile witnesses. (Manager 1)

This lengthy process also gave rise to secondary problems regarding withdrawal of statements and perpetrators (lack of) access to rehabilitation during this period. Support workers described a distinct reason for withdrawal by victim/survivors was through manipulation and intimidation by perpetrators, encouraging victim/survivors to withdraw their statements or not give evidence in court. The length of time perpetrators had to encourage withdrawal before trial was described by support workers as co-occurring with a lack of sufficient safety around the process, including protection orders, bail, and court orders and consistency in how breaches are applied.

Yes, that's right, they were just in the community. And there has been I think nine months between when the incident occurred and when they actually went to court. So, they had along build-up before they went for a trial at least. We had a very long build-up where they could coerce the client to come back into the relationship. (Social worker 1)

Furthermore, as many perpetrators await trial without bail on remand, some support workers described these lengthy waits as contributing to a lack of engagement with perpetrators as they cannot attend relevant perpetrator programs until they have been sentenced. For this group, if sentenced, much of their time will already be served and most would not have the time available to attend programs before release. This was seen as increasing the risk that perpetrators would be released without intervention and seek retribution on victims:

They get put away, put into prison, no further education about their behaviors or intervention. Eventually they are going to be released. So, they’re there stewing over what's occurred. What does that mean for the woman, children, and the society once they’re released without any interventions occurring? (Court support worker 2)

The consequences of the lengthy and often confusing trial process meant safety planning with victim/survivors, including managing contact with perpetrators and potential for future violence and retribution fell back on support workers.

##### Managing Expectations of Sentence Responses

The length of sentences handed down to perpetrators was described by some support workers as insufficient. These support workers felt sentences were too lenient considering the significant and serious act of violence and the continued physical and emotional cost to victim/survivors:

It's all of this stuff she's got to go through because it's full-on, dealing with the police and having to go through all of those processes. Then it's just like, oh, yes, there you go. Serve six months, see you later. It's just so disappointing. (Counselor 7)

Support workers also described feeling frustrated with the inconsistency in which sentencing seemed to be applied, with some noting that they did not know how much more serious the strangulation could be to reach a sentence higher than 3 years. They perceived some victim/survivors as feeling that their perpetrator being charged and sentenced to jail would be a forgone conclusion. Because of this, support workers often described working with victim/survivors to prepare them for an outcome that may disappoint; that if a perpetrator is charged and subsequently sentenced, they may not receive a long jail term:

I have conversations with women after, and they’ll say he’ll go to jail for sure this time. I’ll often say to her, but what if he doesn’t? What is this going to mean for you? (Counselor 6)

## Discussion

In an environment of increasing criminalization of DV in Queensland and internationally, there has been little attention on how these changes have impacted workers in the sector. This qualitative research used the non-fatal strangulation offense as a case study examining support workers’ perceptions of its impact on how they engage with disclosures of strangulation, challenges posed supporting victim/survivors with the offense, and their experiences engaging with criminal law. Our results found that support workers described an overarching experience of heavy and complex workloads related to nonfatal strangulation as a newly criminalized offense that specifically expanded their safety work with victim/survivors limiting their capacity to support freedom work (Woodlock et al., 2023).

These complex and heavy workloads included new service generated and ad hoc strategies to generate knowledge understanding experiences, educate internally and externally, manage victim/survivor expectations, interact and support victim/survivors with criminal legal systems, and attempt to document evidence. Although some of these strategies were likely adapted and expanded from other criminalized behaviors, much of the knowledge around strangulation risk in DV is comparatively new when compared with other types of DV. Therefore, strategies that may apply to other forms of DV would likely be ineffective where knowledge across police, health services, other external services, and the community are each operating with a dearth of information on how to respond to reports of strangulation. Our results suggest that this lack of information and training on strangulation across community and government sectors following criminalization has delayed appropriate knowledge transfer and application. This lack of knowledge in how to respond to strangulation across the spectrum of DV-related services has ultimately overburdened DV workers with the responsibility of education and reduced their capacity to effectively support victim/survivors of strangulation while they do so.

A lack of training delivered in advance of legislative change has resulted in support workers applying a number of flexible and expanding ad hoc professional judgments around strangulation. These included how to ask questions and interpret information around strangulation based on victim/survivors’ understanding, language, and perception of its seriousness that are not currently within validated risk assessments (Douglas & Belfrage, 2015). Training was seen as critical to inform professional judgments for each of these expanding responses to strangulation. However, training itself often created a compounded workload burden, where workers gained responsibility to attend training courses and to then train and mentor colleagues who could not. Furthermore, support workers were also tasked with passing that knowledge on to external services where necessary to advocate for victim/survivors. This included developing and utilizing template letters to health services to suggest directions for screening and evaluation, ensuring there is documented evidence for future prosecution. This indicates that DV services were a site of knowledge production and played an important role in information dissemination.

Training was seen as highly favored to improve service provision to victim/survivors. However, no one discussed receiving training regarding the specific nonfatal strangulation legislation and what to expect from police and court processes. This culminated in several workers not knowing the details of the offense and affecting support workers’ perceptions of how criminal legal processes were functioning. Support workers often reported a sense of frustration with how inconsistent the operation of evidence collection, charges, and court processes seemed. Because of this perceived unpredictability, support workers felt it was their responsibility to ensure evidence was recorded and felt a higher burden navigating information through each process. The length of court processes was not seen as justifying this burden for victim/survivors or themselves for the short (compared to the maximum 7 years) sentences handed down to perpetrators, if they made it to sentencing at all. This seeming unpredictability affected their clients. In particular, support workers emphasized the emotional labor they undertook with victim/survivors across this process as well as through attempts to temper victim/survivors’ expectations of success. Indeed, the length and confusion of this process likely contributes to withdrawals, where previous analysis has found 41% of complainants withdrew from prosecution of nonfatal strangulation in Queensland (Sharman et al., 2022).

Overall, these findings suggest that each new expansion of criminalization is likely to place new and significant long-term burden on support workers as they actively fill the gaps left by governments in supporting victim/survivors and external services with legislative change. The resourcing required for these roles following the introduction of new legislation is thus substantial. However, despite strong government and community reliance on these services, there is no evidence that the impact of expanded criminalization on third sector services is routinely considered. Likely ramifications of this under resourcing are that support workers are contending with the operation of criminal legal systems and educating external services whilst losing time that could be spent on victim/survivors and actively engaging in freedom work. These increasing workloads in the absence of support are also likely to lead to burnout among support workers (Alarcon, 2011). Among our participants this was often represented as disillusionment in the criminal legal response due to stressors related to perceived ambiguity of charging. Further this disillusionment was amplified by heavy workloads related to supporting victim/survivors through long and complex court processes. The introduction of new criminal offenses requires consideration of appropriate funding and resourcing that ensures support workers are equipped with the knowledge and tools they need to deliver support to victim/survivors. A realistic assessment of this resourcing should be factored into the cost of expanding DV legislation. Our participants reported that, in Queensland, there were no mandatory, or even optional, government provided cross-sector training to assist with understanding new legislation. The absence of this likely contributes to support workers frustration with the criminal legal system and ultimately works to the detriment of supporting victim/survivors.

### Third Sector Implications

While we discuss these findings in relation to DV services, these challenges are a likely feature across the third sector (Taylor & Roth, 2019). Health and social services, in particular, are often at the whim of constantly changing policies and legislation. The devolution of government responsibility in these sectors means an increasing reliance on the third sector to fill the gaps of service provision while funding remains scarce and competitive. Workers across the third sector experience similarly expanding workloads while also needing to respond to the ever-changing policies and legislation relevant to their workplaces often without any extra support or funding to do so. It is likely that the issues highlighted by our participants play out in other contexts including welfare, disability services, and aged care to name a few. Thus, findings from this research should be applied more broadly to the third sector to ensure adequate resourcing and training is available to support the diverse policy and legislative transitions that take place in each industry.

In addition, this research also identifies that the third sector can, and often does, play an active role in the way policies and laws play out. With increasing government reliance on criminalization in relation to DV harms, whether and how new laws are interpreted or handled by the third sector, who are often first responders, will significantly impact how criminalization is realized (Baker & McGuirk, 2019). From participants’ comments, it was clear that the Queensland sector is embedded in and works in productive ways to influence other branches of the system (e.g., informing police and doctors about strangulation injuries). Furthermore, their information and support to victim/survivors impacted whether and how they chose to proceed, attend police stations, follow through with evidence collection, and remain engaged with court processes. So, while our results demonstrate the burden on the DV services sector caused by increasing criminalization, they also underscore the importance of the DV sector in the system more broadly given their active influence on the direction, implementation and follow through of government policy (Baker & McGuirk, 2019).

### Limitations

Our study is the first to identify the impact of DV criminal expansion on frontline support workers in the DV sector. However, this research was limited to one state and most services were situated in metropolitan areas. Although some support workers located in regional and rural locations were included, they only accounted for 22% (*n* = 6) of interviewees outside of greater Brisbane and Gold Coast. Given this representation and the geographical diversity of the state of Queensland and Australia as a whole, it is unclear if our findings are generalizable to nonmetropolitan areas. We suspect the problems highlighted by participants are more pronounced in regional and rural areas and some unintended consequences for rural and regional support workers are unlikely to be captured by the data. Similarly, support workers rarely mentioned working with victim/survivors who identified as Aboriginal, Torres Strait Islander, LGBTQ+, or clients with disabilities. We expect further exploration focusing on support work with these victim/survivors would reveal additional difficulties with disclosure, training, and criminal legal interactions that were not explored here. Last, due to the complexity and diversity of the themes found in our analysis, this paper was only able to provide a broader and overarching view of the themes. The research provides a foundation for more detailed analysis in future regarding DV/third sector responses to a new offense.

## Conclusion

DV support workers provide a critical service as a primary avenue into formal support for victim/survivors of DV. Their advocacy is instrumental to assist victim/survivors of violence in whatever direction they choose to take. However, DV support workers’ roles continue to expand in scope where new DV crimes are introduced, yet they receive little extra funding and training to assist in their knowledge. This research explored the hidden impacts of criminal law expansion on support workers, finding substantial increased burdens following the introduction of a new non-fatal strangulation offense. Following the introduction of the offense, support workers described having to learn how the new legislation operated, the medical sequelae of strangulation, and pathways through the criminal legal system while simultaneously educating victim/survivors and aligning external services about these matters. This work was in addition to and compounded upon the work they already engaged in regarding other criminal law responses to DV.

Expansion of criminal laws responding to DV is likely to continue, particularly with discussions around the criminalization of coercive control that could exponentially impact the workload of almost every disclosure of DV (Wangmann, 2022). Considering this, states should consider cross-service legislative training and collaboration so impacted services can better understand new DV offenses, provide expertise across impacted services for police, health, and DV support workers, and avoid overreliance on an already overburdened DV service sector. Furthermore, extra resourcing of these services should be costed into the introduction of new offenses and criminal law changes. Appropriate costing may influence the approach to law reform, while appropriate resourcing would reduce the burden placed on service workers to process and disseminate knowledge about new crimes. This would allow for services to increase capacity and expertise where required and increase the facility for freedom of work.
